# Deubiquitinase Activities Required for Hepatocyte Growth Factor-Induced Scattering of Epithelial Cells

**DOI:** 10.1016/j.cub.2009.07.040

**Published:** 2009-09-15

**Authors:** Richard Buus, Monica Faronato, Dean E. Hammond, Sylvie Urbé, Michael J. Clague

**Affiliations:** 1 Physiological Laboratory, School of Biomedical Sciences, University of Liverpool, Crown Street, Liverpool L69 3BX, UK

**Keywords:** CELLBIO

## Abstract

The scattering response of epithelial cells to activation of the Met receptor tyrosine kinase represents one facet of an “invasive growth” program [1, 2]. It is a complex event that incorporates loss of cell-cell adhesion, morphological changes, and cell motility. Ubiquitination is a reversible posttranslational modification that may target proteins for degradation or coordinate signal transduction pathways [3, 4]. There are ∼79 active deubiquitinating enzymes (DUBs) predicted in the human genome [5, 6]. Here, via a small interfering RNA (siRNA) library approach, we have identified 12 DUBs that are necessary for aspects of the hepatocyte growth factor (HGF)-dependent scattering response of A549 cells. Different phenotypes are evident that range from full loss of scattering, similar to receptor knockdown (e.g., USP30, USP33, USP47), to loss of cell-cell contacts even in the absence of HGF but defective motility (e.g., USP3, ATXN3L). The knockdowns do not incur defective receptor, phosphatidylinositol 3-kinase, or MAP kinase activation. Our data suggest widespread involvement of the ubiquitin system at multiple stages of the Met activation response, implying significant crosstalk with phosphorylation-based transduction pathways. Development of small-molecule inhibitors of particular DUBs may offer a therapeutic approach to contain metastasis.

## Results and Discussion

**Figure 1:**
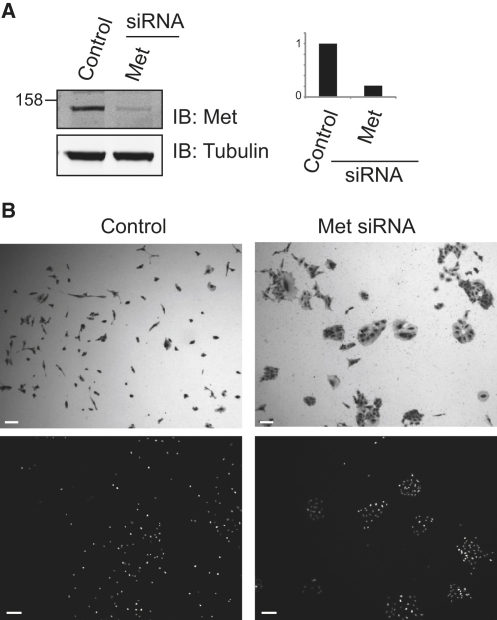
Inhibition of HGF-Induced Scattering Response of A549 Cells by siRNA Knockdown of the Met Receptor (A) Reduction in cellular Met receptor levels following incubation with siRNA oligos directed against the Met receptor. (B) A549 cells treated with vehicle (Oligofectamine, left panels) or Met siRNA (right panels) and stimulated with 50 ng/ml hepatocyte growth factor (HGF) for 12 hr. Cells were fixed and stained with crystal violet (top panels) or DAPI (bottom panels). Scale bars represent 100 μm.

We have grown lung adenocarcinoma A549 cells under conditions where they form small islands typically consisting of 10–25 cells. Upon stimulation with hepatocyte growth factor (HGF), the cells scatter over a 12–16 hr time period to produce a largely dispersed field of single motile cells, which we fix and stain with crystal violet to enhance contrast for light microscopy. Alternatively, cells can be visualized by fluorescence microscopy following DAPI staining. This process is inhibited by knockdown of the Met receptor (Figure 1). We used these assays to test for a role of deubiquitinating enzymes (DUBs) in regulating the HGF scattering response. A549 cells were depleted of specific DUBs with small interfering RNAs (siRNAs) for 85 human DUB genes (see Table S1 available online) comprising a pool of four oligonucleotides targeting unique sequences in each gene (siGenome library, Dharmacon). Effects upon HGF-induced cell scattering were then observed by light microscopy. Three repetitions of this screen were examined by three observers each time and independently scored for inhibition of scattering. This produced a consensus list of 13 candidate DUBs (∼15% of the total) drawn from 4 of the 5 DUB families (no JAMM DUBs were identified).

To assess the prevalence of off-target effects, we deconvoluted the oligonucleotide pools, which must contain at least one inhibitory component, for each DUB candidate. If our screen simply reflected nonspecific off-target inhibitory effects, statistical considerations predict that only one or two of the 13 deconvoluted oligonucleotide pools would contain a second inhibitory oligonucleotide. For 12 of the 13 targets, at least two oligonucleotides effectively inhibited HGF-dependent scattering. The exception, UCHL5, was not pursued further. In all, 35 of 52 oligos tested in these validation experiments produced clearly discernible inhibitory effects (Table S2; Figure S1).

**Figure 2:**
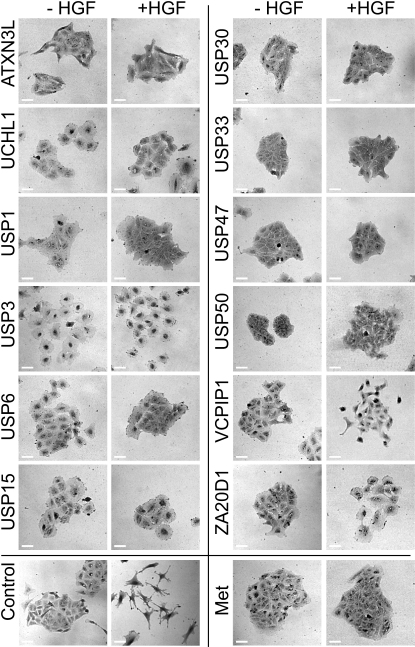
Morphological Features of A549 Cells following Selected DUB Knockdown Twelve deubiquitinating enzymes (DUBs) identified in our screen as required for HGF-mediated scattering of A549 cells were knocked down with pooled oligonucleotides from a siGenome library. One set of cells was treated with 50 ng/ml HGF for 12 hr (+HGF panels), while the other set was left untreated (−HGF panels). Cells were stained with crystal violet. Scale bars represent 50 μm.

Within the group of 12 targets, we could observe different phenotypic outcomes, which we separated broadly into three classes (Figure 2): (1) large, flat cells (ATXN3L, UCHL1, USP3, USP6, USP15, ZA20D1/Cezanne); (2) cells in which cell-cell contacts had largely broken down but the cells remained clustered (USP50, VCPIP1); and (3) cells remaining in tight clusters similar to unstimulated or Met-depleted cells (USP1, USP30, USP33, USP47). The class 1 phenotype was independent of HGF stimulation (Figure 2) in all cases. We believe that this reflects a general decrease in the motility of the cells, such that they cannot move apart following HGF stimulation although other aspects of the program may remain intact. In the case of USP3 knockdown, cell-cell contacts were almost completely lost prior to addition of HGF. Perhaps most interesting is the class 3 phenotype, which is barely distinguishable from knockdown of the Met receptor itself.

We examined the appearance of unstimulated cells following each DUB knockdown with each of the individual oligos from the initial pool. One interesting finding was that in several instances, this led to a scattered phenotype (Table S2; Figure S2), which is recessive in the context of the pool. In all cases, the morphological appearance characteristic of knockdown by pooled oligonucleotides was found with ≥2 individual oligonucleotides.

**Figure 3:**
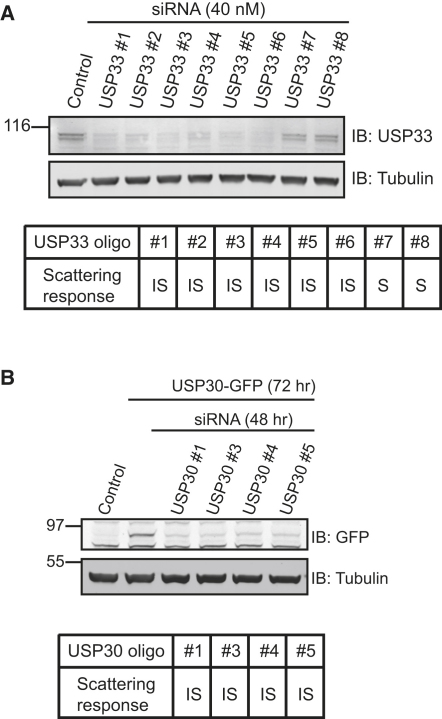
Correlation of USP33 and USP30 Knockdown Efficiency with HGF-Induced Scattering Response in A549 Cells (A) Estimation by western blotting of endogenous protein of the knockdown efficiency for eight individual siRNA oligonucleotides directed against USP33 (top) and their respective effects on HGF-mediated cell scattering of A549 cells (bottom) (IS, inhibition of scattering; S, scattering). (B) No antibody is currently available for USP30, but each of the four individual oligonucleotides effectively represses transient expression of USP30-GFP in HeLa cells as judged by western blotting with GFP-antibody, as well as inhibiting HGF-mediated scattering of A549 cells (IS).

For some DUBs, we were able to judge the extent of knockdown by using antibodies that recognize the endogenous protein (UCHL1, USP1, USP15, USP33, USP47). With the exception of USP1, there was a good correlation with knockdown efficiency. A striking example is USP33, for which we have now used a total of eight individual oligos, adding four On-Target Plus oligos to the original four from the siGenome pool. Figure 3A illustrates the knockdown efficiency of USP33 and the strong correlation with scattering inhibition. In the case of the mitochondrial DUB USP30 [7], we have validated that all four oligos, which inhibit scattering, effectively suppress the expression of transfected USP30-GFP (Figure 3B) and endogenous mRNA levels (data not shown).

**Figure 4:**
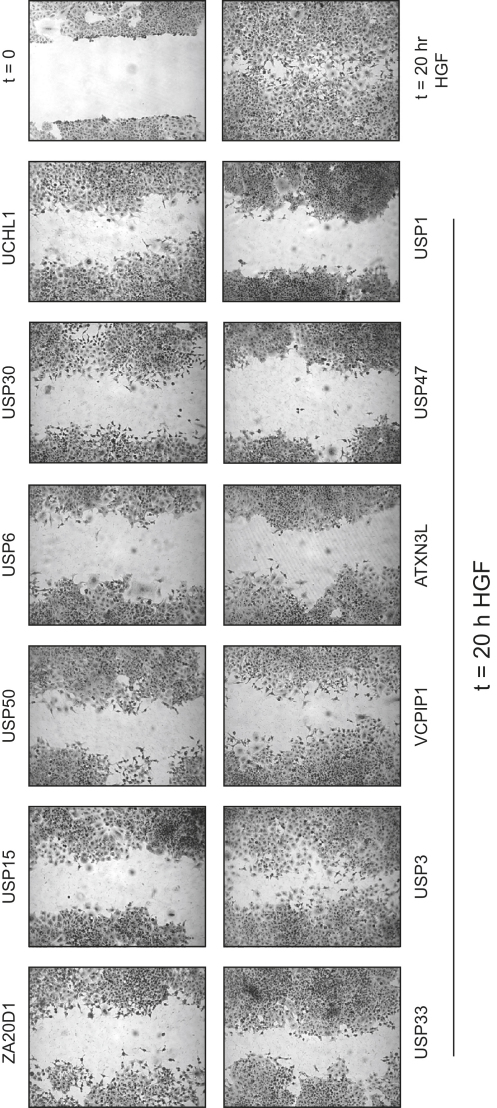
DUB Requirements for HGF-Dependent Wound Healing in A549 Cells Confluent monolayers of A549 cells were pretreated for 72 hr with siRNA oligonucleotides (40 nM) consisting of pools from the siGenome DUB library targeting specific DUBs. After introducing a scratch, cells were incubated in full medium supplemented with 50 ng/ml HGF and visualized 20 hr later.

We next performed another assay reflecting cell invasion, the wound-healing assay, which monitors the HGF-stimulated migration of cells into a scratch made in a confluent monolayer of A549 cells. Again, we observed that all DUBs identified in our scattering screen, with the exception of USP3, also inhibited this type of assay (Figure 4). Similar results were obtained with the human pancreatic Panc1 cell line, the exceptions here being USP6 and USP47 (Figure S3).

The most trivial explanation of these results could be that scattering inhibition might reflect defects in receptor activation or in one of the canonical signaling pathways associated with cell scattering. We assayed total cellular Met receptor levels following knockdown of each of the 12 DUBs with the oligonucleotide pools. No major changes were observed, with the exception of USP6, where receptor levels declined by a factor of two (Figure S4A), but this effect could largely be attributed to a single oligonucleotide (Figure S5). The specific activity of Met receptor phosphorylation was assayed by using a phospho-Met-specific antibody, and the characteristic downregulation of receptor that follows acute HGF stimulation was measured after 2 hr [8, 9]. In all cases, the response appeared broadly normal (Figure S4B), indicating that the receptor is initially at the cell surface, becomes activated, and elicits the downstream response necessary for lysosome-directed trafficking of activated receptor [9].

In Madin-Darby canine kidney cells, both MAP kinase and phosphatidylinositol 3-kinase pathways are required for HGF-induced adherens junction disassembly based on sensitivity to pharmacological inhibitors [10]. We examined HGF-dependent phosphorylation of a component of the MAP kinase cascade, ERK, and of PKB/Akt, which is contingent upon phosphatidylinositol 3-kinase activity [11] (Figures S6A and S6B). In all cases, these major signaling pathways were largely intact, with the exception of USP1, for which a 50% reduction in phospho-ERK was observed (Figure S6A). However, this effect of the pooled oligonucleotides did not deconvolute in a manner that correlated with USP1 knockdown efficiency and was not pursued (data not shown). An MTS assay reflecting cell proliferation and viability also indicated no significant effects following DUB knockdown (Figure S6C). Focal adhesion kinase (FAK) is phosphorylated following HGF treatment and stimulates the motility component of the scattering response [12, 13]. No changes in HGF-induced FAK phosphorylation were observed following knockdown of each of the DUB targets, with the exception of USP6, but again, this effect did not deconvolute to more than one individual oligonucleotide (Figure S7).

Most of the DUBs we have identified in this screen are poorly annotated. Notable exceptions are USP33, which is known to interact with von Hippel-Lindau (VHL) protein [14]; ZA20D1/Cezanne, a negative regulator of the NFκB pathway [15]; and USP1, which can deubiquitinate the DNA replication processivity factor PCNA [16] and regulates the Fanconi anemia pathway through deubiquitination of FANCD2 [17]. VCPIP1 interacts with p97, a highly abundant protein implicated in a vast range of cellular activities including the endoplasmic reticulum-associated degradation (ERAD) pathway and HIF1α stability [18]. USP50 is likely to be a catalytically inactive DUB [19]. Most pertinently, overexpression of UCHL1 has been found to correlate with the metastatic phenotype of renal cell carcinomas [20], and USP6/TRE17 has been suggested to be in complex with CDC42 and Rac1 regulating actin rearrangements [21]. USP6 knockdown also leads to decreased activation of Arf6, a GTPase associated with cell invasion [22].

The screen reported here does not assess the requirements for catalytic activity of the DUBs, but it seems reasonable to assume that this will be the case in many instances. To date, the major established role of ubiquitin following receptor tyrosine kinase stimulation has been to promote lysosomal degradation of the receptor [23, 24]. Our data suggest hitherto unappreciated roles of ubiquitin-linked events in regulating the outcomes of HGF signaling, although the relevant substrates are currently unclear. The utility of DUBs as therapeutic drug targets has been discussed, and some first-generation inhibitors of USP7 and USP8 have recently been developed [25]. Several candidate DUBs arising from this screen suggest themselves as novel targets for antimetastasis therapies.

## Experimental Procedures

### Oligonucleotides

A library of oligonucleotides targeting 85 DUBs was purchased from Dharmacon comprising pools of four individual oligonucleotides from their siGenome collection. Individual oligonucleotides for deconvolution, as well as four further oligonucleotides from their On-Target Plus collection in the case of USP33, were also obtained from Dharmacon.

### Antibodies

Antibodies were purchased from the following sources: mouse anti-Met receptor, anti-phospho-Akt, and anti-phospho-MAPK from Cell Signaling; rabbit anti-USP33, anti-USP15, and anti-USP47 from Bethyl Laboratories; rabbit anti-UCHL1 from Abcam; and mouse anti-tubulin from Sigma. Rabbit anti-USP1 was a gift from A. D'Andrea (Harvard University).

### HGF-Dependent Cell Scattering Screen

A549 cells were cultured in Dulbecco's modified Eagle's medium supplemented with 10% fetal bovine serum and 1% nonessential amino acids. Small islands of cells were seeded following limited trypsin digestion of confluent cells and plated at 600 cells per well of a 48-well plate. Sixteen hours later, siRNA oligonucleotides (40 nM) targeting specific DUBs were delivered to cells with Oligofectamine. After 72 hr, cells were treated with 50 ng/ml HGF (gift of G. Vande Woude, Van Andel Research Institute, Grand Rapids, MI) for 16 hr followed by fixation with paraformaldehyde. Cells were then visualized by staining with crystal violet for bright-field microscopy or DAPI for fluorescence microscopy.

### Wound-Healing Assay

A549 cells were seeded in a 12-well plate at 28,000 cells per well. The following day, they were transfected with library-derived pools of oligonucleotides (40 nM) targeting specific DUBs with Oligofectamine. After 72 hr, a linear scratch in the confluent cell monolayer was made with a sterile pipette tip. Cells were rinsed and then incubated in full medium supplemented with HGF for 20 hr prior to fixation followed by staining with crystal violet solution to enhance contrast. For each well, six pictures were taken at 10× magnification along the scratch area with a Leica DFC 350F phase-contrast microscope.

## Supplemental Data

Document S1. Seven Figures and Three Tables
