# Supplementary material for: Deubiquitinase Activities Required for Hepatocyte Growth Factor-Induced Scattering of Epithelial Cells
Source: Curr Biol. 2009 Sep 15;19(17):1463–6. doi: 10.1016/j.cub.2009.07.040 (PMC2764384; doi:10.1016/j.cub.2009.07.040)
Supplement: Document S1. Seven Figures and Three Tables [file mmc1.pdf]

Current Biology, Volume *19*

Supplemental Data

Deubiquitinase Activities Required for Hepatocyte Growth Factor-Induced Scattering of Epithelial Cells

Richard Buus, Monica Faronato, Dean E. Hammond, Sylvie Urbé, and Michael J. Clague

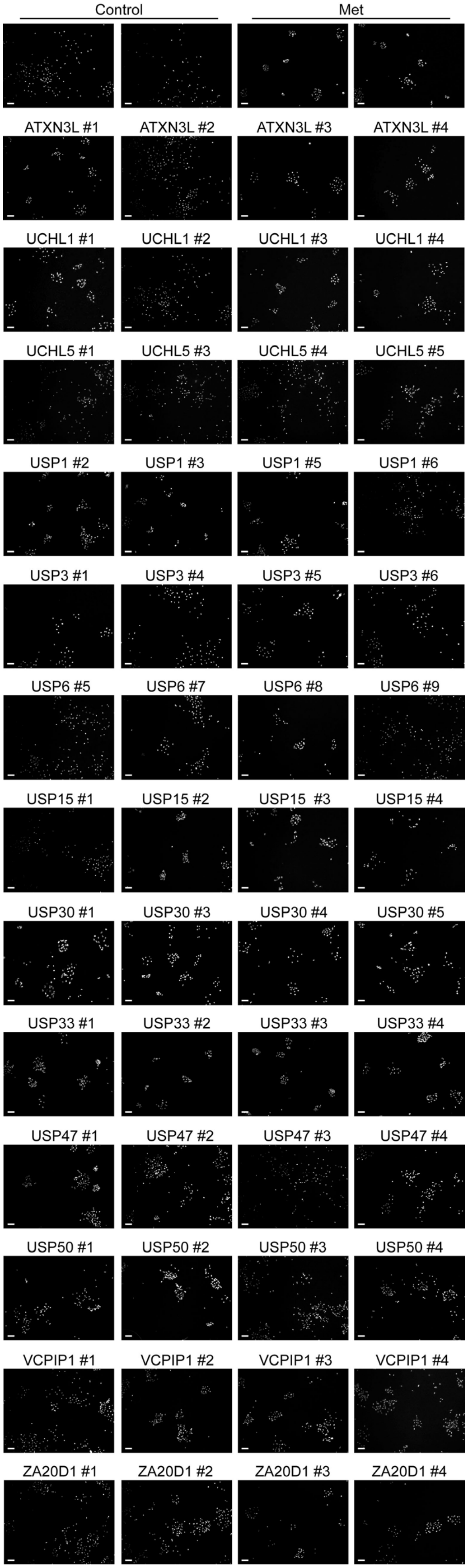

Figure S1. Deconvolution of Oligonucleotide Pools Used in the Initial Screen of the HGF-Dependent Cell Scattering Response

A549 cells were treated for 72 hours with individual oligonucleotides derived from the original pool of four, used in the initial screen of HGF-dependent cell scattering. Cells were then treated with 50ng/ml HGF overnight, then fixed and stained with DAPI. Representative images are shown along with the Oligofectamine only (Control) and Met knockdown controls. Scale bar represents 100 μm. Results are collated in Table S2.

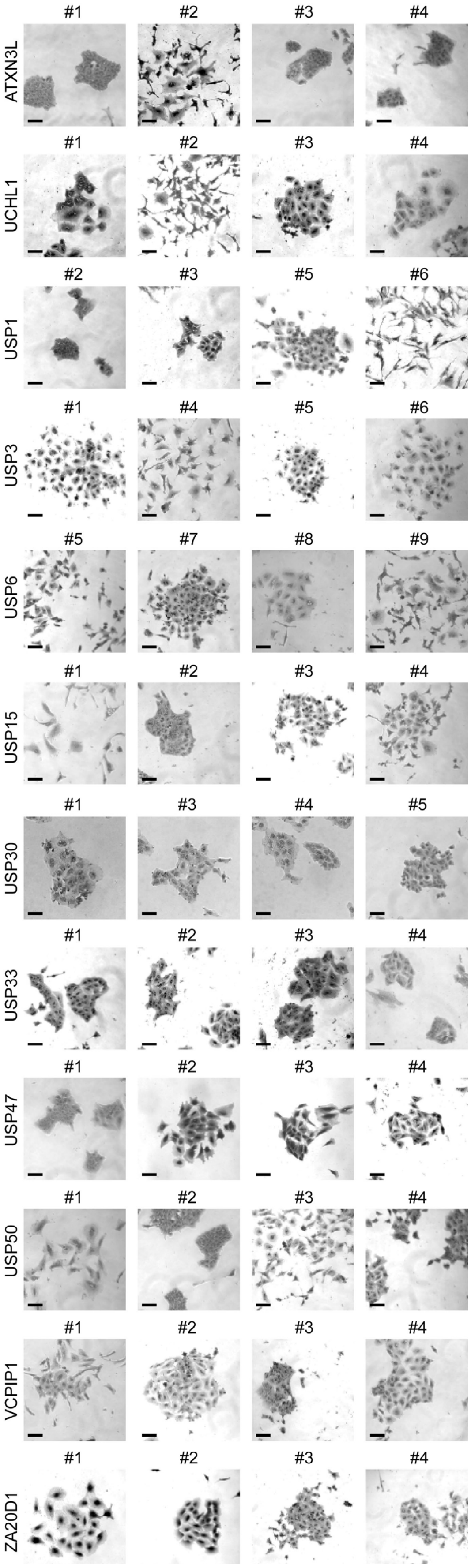

Figure S2. Deconvolution of Oligonucleotide Pools for Morphological Changes

A549 cells were incubated for 72 hours with individual oligonucleotides derived from the original pool of four, used in the initial screen of HGF-dependent cell scattering. Cells were then stained with crystal violet for clearer visualization. Representative images are shown. Scale bar represents 50 μm. Results are collated in Table S3.

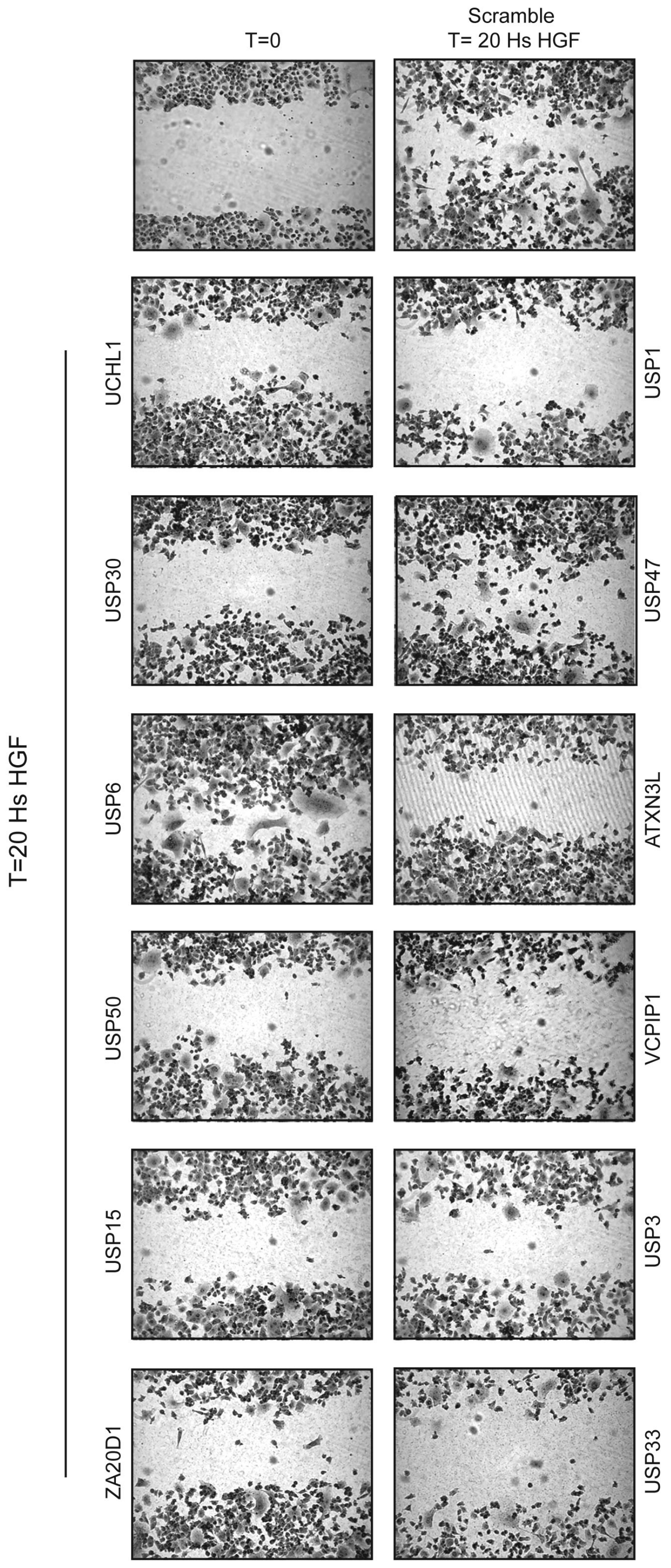

Figure S3. DUB Requirements for HGF-Dependent Wound Healing in Panc1 Cells

Confluent monolayers of Panc1 cells were pre-treated for 72 hr with siRNA oligonucleotides consisting of pools from the siGenome DUB library targeting specific DUBs. After introducing a scratch, cells were incubated in full medium supplemented with 50ng/ml HGF and visualized 20 hr later.

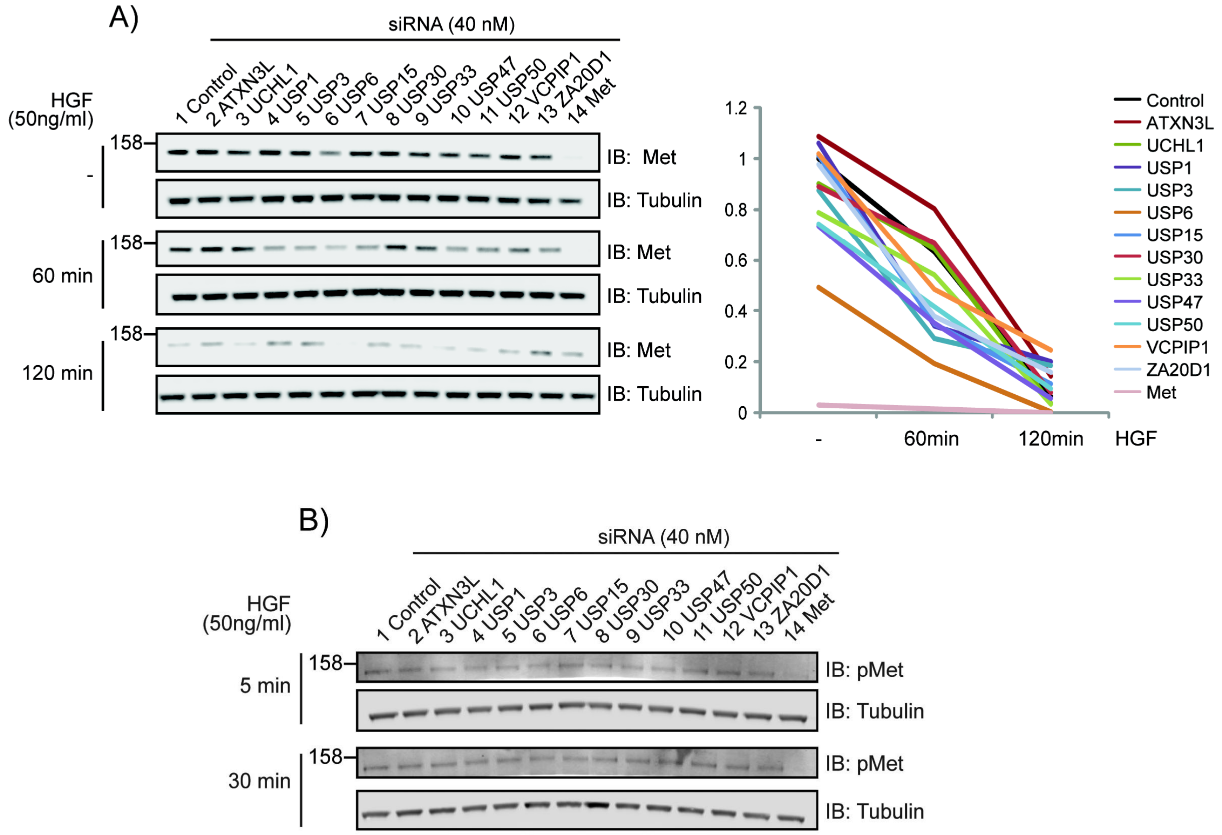

Figure S4. Effect of DUB Knockdown on Met Receptor Levels and HGF-Dependent Phosphorylation of Receptor

(A) Met receptor levels in cells depleted of twelve DUBs identified in the scattering screen, normalized to unstimulated control cells (treated with Oligofectamine). 50ng/ml of HGF was applied for the indicated times.

(B) Phosphorylation of Met receptor (pTyr1349), 5 and 30 minutes post HGF stimulation and knockdown of specific DUBs or Met receptor (Lane 14).

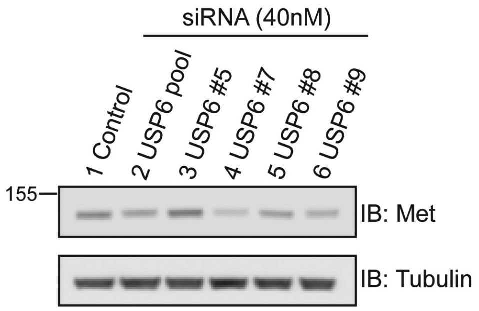

Figure S5. Deconvolution of Oligonucleotides Targeting USP6 for Effects on Met Receptor Levels in A549 Cells

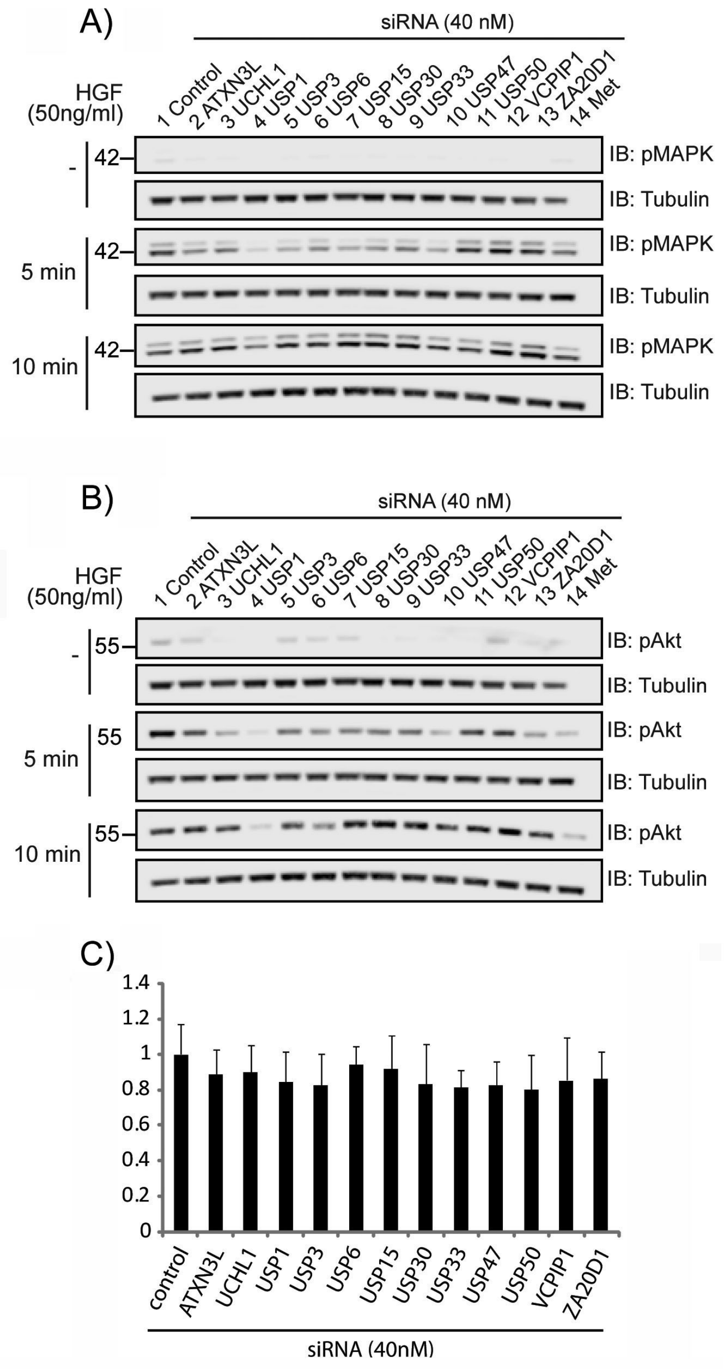

Figure S6. Phosphorylation of PKB/Akt and MAPK following HGF Stimulation as Well as Cell Viability Are Unaffected by DUB Knockdown

(A and B) Downstream signals in A549 cells, stimulated for 5 and 10 minutes with 50ng/ml HGF assessed by antibodies recognising (A) phospho-MAPK (pT202, pY204) and (B) phospho-Akt (pS473).

(C) Effect of DUB knockdown on cell viability determined by an MTS assay, which measures cellular respiration. Error bars correspond to standard deviation.

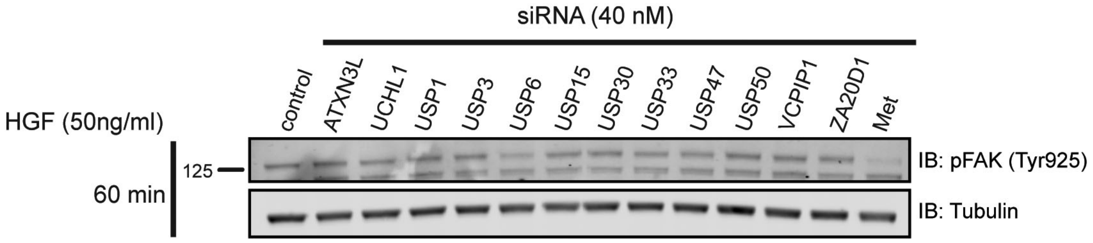

Figure S7. HGF-Dependent Phosphorylation of Focal Adhesion Kinase (FAK) Is Not Affected by siRNA-Mediated Knockdown of Twelve DUBs

A549 cells were treated with pools of oligonucleotides (siGenome) targeting twelve DUBs implicated in HGF-dependent cell scattering. Cells were stimulated for 60 minutes with 50ng/ml HGF.

Table S1. List of DUB Targets Included in the HGF Scattering Screen

Gene Number	Gene Symbol
1	*CYLD*
2	*DUB1A*
3	*DUB3*
4	*DUB4*
5	*OTUB1*
6	*OTUB2*
7	*PSMD14*
8	*UCHL1*
9	*UCHL3*
10	*UCHL5*
11	*USP1*
12	*USP10*
13	*USP11*
14	*USP12*
15	*USP13*
16	*USP14*
17	*USP15*
18	*USP16*
19	*USP18*
20	*USP19*
21	*USP2*
22	*USP20*
23	*USP21*
24	*USP22*
25	*USP24*
26	*USP25*
27	*USP26*
28	*USP28*
29	*USP29*
30	*USP3*

31	*USP30*
32	*USP31*
33	*USP32*
34	*USP33*
35	*USP34*
36	*USP35*
37	*USP36*
38	*USP37*
39	*USP38*
40	*USP39*
41	*USP4*
42	*USP40*
43	*USP41*
44	*USP42*
45	*USP43*
46	*USP44*
47	*USP45*
48	*USP46*
49	*USP47*
50	*USP48*
51	*USP49*
52	*USP5*
53	*USP50*
54	*USP51*
55	*USP52*
56	*USP53*
57	*USP54*
58	*USP6*
59	*USP7*
60	*USP8*
61	*USP9X*
62	*USP9Y*
63	*BAP1*
64	*ATXN3L*
65	*JOSD1*
66	*TNFAIP3*
67	*ZA20D1*
68	*OTUD7*
69	*OTUD4*
70	*YOD1*
71	*OTUD6B*
72	*OTUD5*
73	*OTUD1*
74	*ZRANB1*
75	*VCPIP1*
76	*PARP11*
77	*STAMBP*
78	*STAMBPL1*
79	*CXORF53*
80	*COPS5*
81	*FLJ14981*
82	*MYSM1*
83	*PRPF8*
84	*MJD* (*ATXN3*)
85	*SBBI54* (*JOSD2*)

Table S2. Summary of Deconvolution Data from Figure S1

	Four oligos				Number of oligos showing scattering inhibitory effect
**ATXN3L**	**IS**	S	**IS**	**IS**	3
**UCHL1**	**IS**	S	**IS**	**IS**	3
**UCHL5**	S	S	S	**IS**	1
**USP1**	**IS**	**IS**	**IS**	S	3
**USP3**	**IS**	S	**IS**	**IS**	3
**USP6**	S	**IS**	**IS**	S	2
**USP15**	S	**IS**	**IS**	**IS**	3
**USP30**	**IS**	**IS**	**IS**	**IS**	4
**USP33**	**IS**	**IS**	**IS**	**IS**	4
**USP47**	**IS**	S	S	**IS**	2
**USP50**	S	**IS**	S	**IS**	2
**VCPIP1**	S	**IS**	**IS**	**IS**	3
**ZA20D1**	S	S	**IS**	**IS**	2

Table S3. Phenotypes Observed following Depletion of the Twelve DUBs with Individual Oligos

**Symbol**	**Pool**	**1st oligo**	**2nd oligo**	**3rd oligo**	**4th oligo**
**ATXN3L**	T	T	D	T	T
**UCHL1**	F	F	F, D	F	F
**USP1**	T	T	T	F	D
**USP3**	F	F	D	F	F
**USP6**	F	F, D	F	F	F, D
**USP15**	F	D	T	F	F
**USP30**	T	T	T	T	T
**USP33**	T	T	T	T	T
**USP47**	T	T	F	F	T
**USP50**	T	F, D	T	F, D	T
**VCPIP1**	T	F	F	T	F
**ZA20D1**	F	F	F	F	F

Characteristic morphologies observed following knockdown of DUBs with four individual oligonucleotides (Figure S2) compared with the pool (Figure 2). Abbreviations: D: dispersed cells; F: flattened cells; T: tightly packed islands.
